# Supplementary material for: Type I IFN exacerbates disease in tuberculosis-susceptible mice by inducing neutrophil-mediated lung inflammation and NETosis
Source: Nat Commun. 2020 Nov 4;11:5566. doi: 10.1038/s41467-020-19412-6 (PMC7643080; doi:10.1038/s41467-020-19412-6)
Supplement: Supplementary file 1 — Supplementary Information [file 41467_2020_19412_MOESM1_ESM.pdf]

Supplementary Information

Type I IFN exacerbates disease in tuberculosis-susceptible mice by inducing neutrophil-mediated lung inflammation and NETosis

Lúcia Moreira-Teixeira[1,*], Philippa J. Stimpson[1], Evangelos Stavropoulos[1], Sabelo Hadebe[1], Probir Chakravarty[2], Marianna Ioannou[3], Iker Valle Aramburu[3], Eleanor Herbert[4,5], Simon L. Priestnall[4,5], Alejandro Suarez-Bonnet[4,5], Jeremy Sousa[6,7,8], Kaori L. Fonseca[6,7,8,9], Qian Wang[3], Sergo Vashakidze[10], Paula Rodríguez-Martínez[11], Cristina Vilaplana[12], Margarida Saraiva[6,7,#], Venizelos Papayannopoulos[3,#] and Anne O'Garra[1,13,#]

[1]Laboratory of Immunoregulation and Infection, The Francis Crick Institute, London NW1 1AT, UK.

[2]Bioinformatics and Biostatistics Team, The Francis Crick Institute, London NW1 1AT, UK.

[3]Laboratory of Antimicrobial Defence, The Francis Crick Institute, London NW1 1AT, UK.

[4]Department of Pathobiology and Population Sciences, Royal Veterinary College, Hatfield AL9 7TA, UK.

[5]Experimental Histopathology Team, The Francis Crick Institute, London NW1 1AT, UK.

[6]i3S - Instituto de Investigação e Inovação em Saúde, Universidade do Porto, Portugal.

[7]IBMC - Instituto de Biologia Molecular e Celular, Universidade do Porto, Porto, Portugal.

[8]ICBAS - Instituto de Ciências Biomédicas Abel Salazar, Universidade do Porto, Porto, Portugal.

[9]Programa de Pós-Graduação Ciência para o Desenvolvimento (PGCD), Instituto Gulbenkian de Ciência (IGC), Oeiras, Portugal.

[10]National Center for Tuberculosis and Lung Diseases (NCTLD), 50, Maruashvili Str. 0101 Tbilisi, Georgia.

[11]Pathology Department, Hospital Universitari Germans Trias i Pujol, Universitat Autònoma de Barcelona (UAB), Crtra. Del Canyet, s/n. 08916, Badalona, Catalonia; Spain.

[12]Experimental Tuberculosis Unit (UTE), Fundació Institut Germans Trias i Pujol (IGTP), Universitat Autònoma de Barcelona (UAB), CIBER Enfermedades Respiratorias. Edifici Laboratoris de Recerca. Can Ruti Campus. Crtra. de Can Ruti, Camí de les Escoles, s/n. 08916, Badalona, Catalonia; Spain.

[13]National Heart and Lung Institute, Faculty of Medicine, Imperial College London, London W2 1PG, UK.

*Correspondence and requests for materials should be addressed to Lúcia Moreira-Teixeira, e-mail: luciamoreirateixeira@gmail.com

#These authors jointly supervised this work.

Supplementary Figure 1

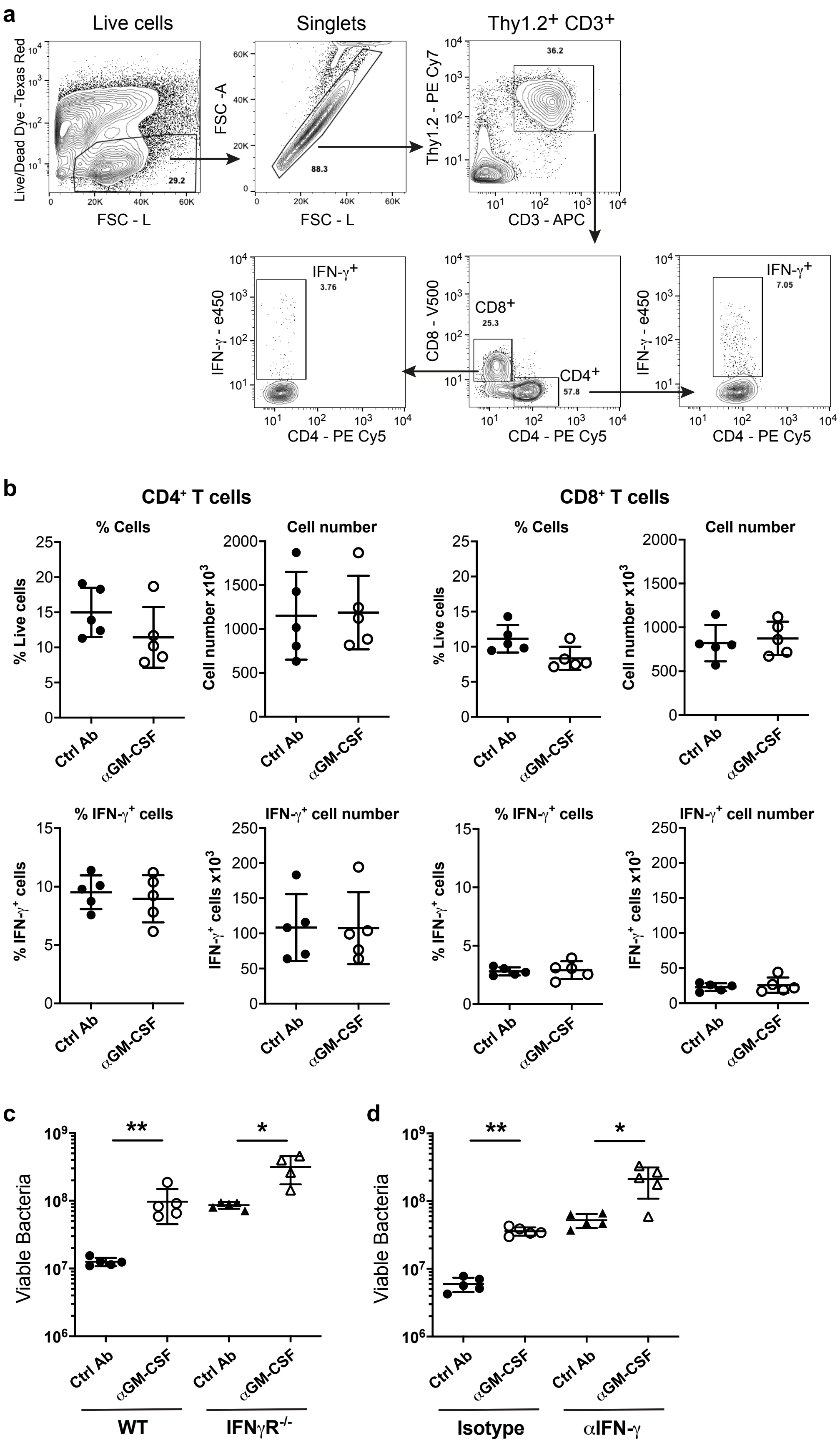

Supplementary Figure 1. Disease exacerbation during *M. tuberculosis* infection resulting from GM-CSF blockade does not require IFN-γ. a, b, WT mice were infected and treated with Ctrl Ab (closed circles) or αGM-CSF (open circles) as in Fig. 1a. At day 21 post-infection, lung cell suspensions were prepared and stained for detection of T cells. Production of IFN-γ was determined after *ex vivo* restimulation of whole lung cell suspensions with PPD plus anti-CD28 overnight and brefeldin A for the last 4 h. **a**, Gating strategy for quantification of $CD4^+$ and $CD8^+$ T cells, and IFN-γ-expressing T cells is shown. **b**, The percentage of $CD4^+$ (top left) and $CD8^+$ (top right) T cells among total live cells and total cell numbers for both are shown. The percentage of IFN-γ-expressing cells among $CD4^+$ (bottom left) and $CD8^+$ (bottom right) T cells and total cell numbers for both are shown. **c,** WT (circles) or $IFN\gamma R^{-/-}$ (triangles) mice were infected and treated with Ctrl Ab (closed symbols) or αGM-CSF (open symbols) as in Fig. 1a. Lung viable bacterial loads were determined at day 21 post-infection. $^{**}P = 0.0079$; $^{*}P = 0.0159$. **d,** WT mice were infected and treated with Ctrl Ab (closed circles) or αGM-CSF (open circles) as in Fig. 1a with the exception that mice also received 0.5 mg of anti-IFN-γ (αIFN-γ; triangles) or isotype mAbs (circles) with each i.p. injection. Lung viable bacterial loads were determined at day 21 post-infection. $^{**}P = 0.0079$; $^{*}P = 0.0317$. Data representative of five (**b**) or two (**c**, **d**) biological experiments. Represented is the mean±SD; each dot represents an individual mouse: n = 5 mice/group (**b**, **d**) or n = 4-5 mice/group (**c**). Source data are provided as a Source Data file. Statistical analysis was performed using two-tailed Mann-Whitney test.

Supplementary Figure 2

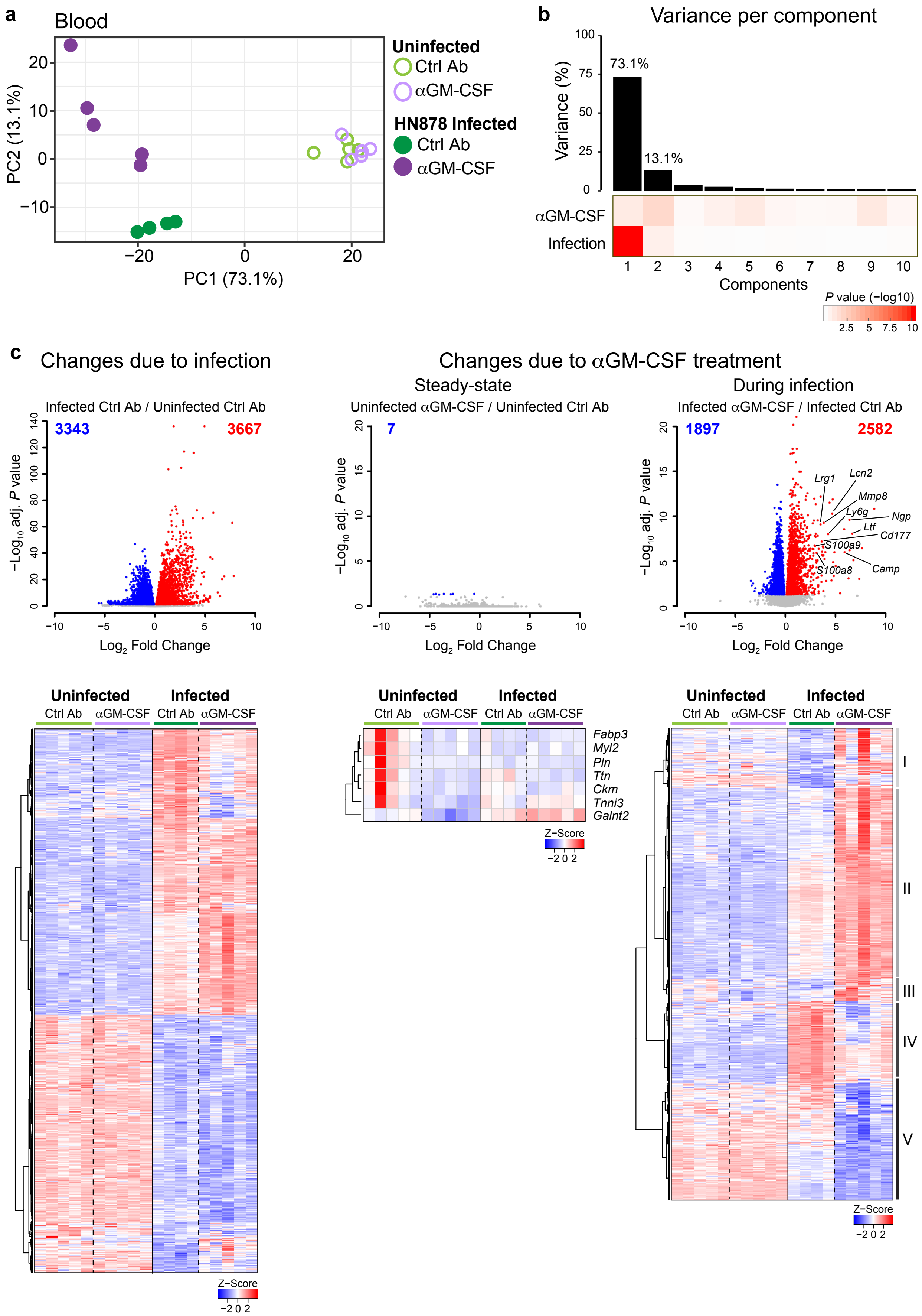

Supplementary Figure 2. Blood transcriptomic changes upon *M. tuberculosis* infection and anti-GM-CSF mAbs treatment. WT mice were infected and/or treated as in Fig. 1a. Blood was collected from infected and uninfected mice for RNA-Seq analysis (n= 4-5 mice/group). **a,** Principal-component (PC) analysis depicting the variation in the global gene expression profiles across the different groups. PC1 and PC2, which capture the greatest variation in gene expression, are shown. Empty and filled symbols represent uninfected and infected samples, respectively; colour represents mAbs treatment (isotype control (Ctrl Ab) in green; αGM-CSF in purple). Each dot represents an individual mouse. **b,** Variance per PC identifying the main sources of variation in gene expression between samples from HN878 infected and uninfected mice (Infection) and/or αGM-CSF and Ctrl Ab treated mice (αGM-CSF). The significance of each principal component was assessed by an F-test of the associated regression model. **c,** Volcano plots (top) and heatmaps (bottom) depicting the differently expressed genes between blood samples from infected Ctrl Ab treated versus uninfected Ctrl Ab treated mice (left), uninfected αGM-CSF treated versus uninfected Ctrl Ab treated mice (middle), infected αGM-CSF treated versus infected Ctrl Ab treated mice (right) (Supplementary Data 2). Volcano plots: numbers and colour relate to genes that have an adjusted P value < 0.05. Blue, significantly down-regulated; red, significantly up-regulated; grey, non-differentially expressed. P values were adjusted for multiple testing using the Benjamini-Hochberg method. Heatmaps: gene expression values were averaged and scaled across the row to indicate the number of standard deviations above (red) or below (blue) the mean, denoted as row Z-score; shown for individual blood samples from uninfected Ctrl Ab (light green) or αGM-CSF (light purple) and infected Ctrl Ab (dark green) or αGM-CSF (dark purple) treated mice. Dendrogram shows unsupervised hierarchical clustering of genes.

Supplementary Figure 3

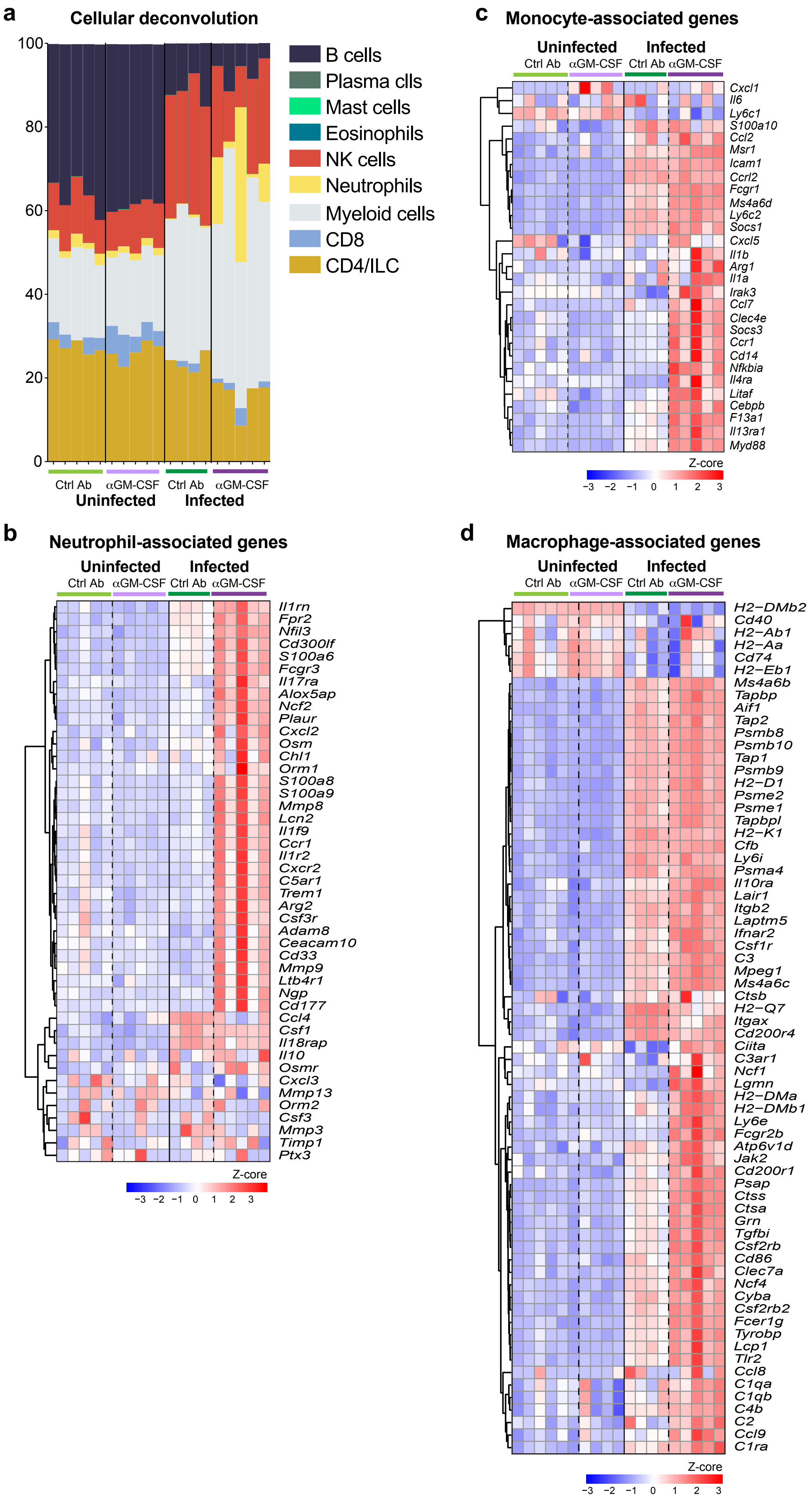

Supplementary Figure 3. Blood transcriptomic changes in myeloid populations upon *M. tuberculosis* infection and anti-GM-CSF mAbs treatment. WT mice were infected and/or treated as in Fig. 1a. Blood was collected from infected and uninfected mice for RNA-Seq analysis (n= 4-5 mice/group). **a,** Stacked bar plots depicting in silico immune cell composition of mouse blood RNA-seq samples, derived using the CIBERSORT algorithm based on cellular signatures obtained from ImmuCC. Each bar represents percent fractions for 9 representative cell types for an individual mouse sample, with colours representing the different cell types. ILC, innate lymphoid cells. Yellow bars indicate neutrophils; grey bars indicate monocytes and macrophages (labelled collectively as myeloid cells which excludes neutrophils). **b-d,** Heatmap showing relative expression of genes in the neutrophil (**b**), and monocyte (**c**) and macrophage (**d**) -associated gene set for individual blood samples from uninfected Ctrl Ab (light green) or αGM-CSF (light purple) and infected Ctrl Ab (dark green) or αGM-CSF (dark purple) treated mice. Gene expression values were averaged and scaled across the row to indicate the number of standard deviations above (red) or below (blue) the mean, denoted as row Z-score. Dendrogram shows unsupervised hierarchical clustering of genes.

Supplementary Figure 4

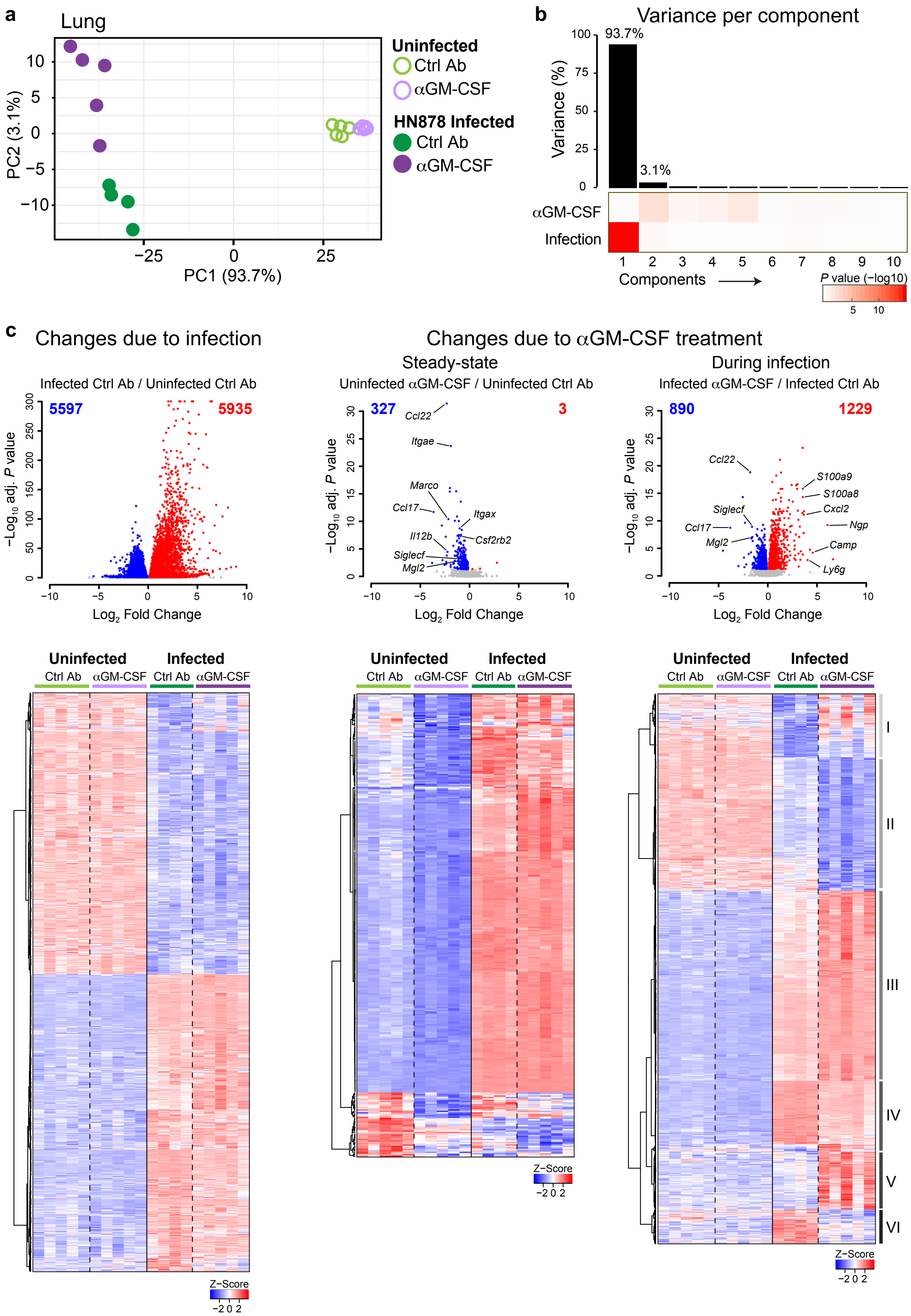

Supplementary Figure 4. Lung transcriptomic changes upon *M. tuberculosis* infection and anti-GM-CSF mAbs treatment. WT mice were infected and/or treated as in Fig. 1a. Lungs were harvested from infected and uninfected mice for RNA-Seq analysis (n= 4-5 mice/group). **a,** Principal-component (PC) analysis depicting the variation in the global gene expression profiles across the different groups. PC1 and PC2, which capture the greatest variation in gene expression, are shown. Empty and filled symbols represent uninfected and infected samples, respectively; colour represents mAbs treatment (isotype control (Ctrl Ab) in green; αGM-CSF in purple). Each dot represents an individual mouse. **b,** Variance per PC identifying the main sources of variation in gene expression between samples from HN878 infected and uninfected mice (Infection) and/or αGM-CSF and Ctrl Ab treated mice (αGM-CSF). The significance of each principal component was assessed by an F-test of the associated regression model. **c,** Volcano plots (top) and heatmaps (bottom) depicting the differently expressed genes between lung samples from infected Ctrl Ab treated versus uninfected Ctrl Ab treated mice (left), uninfected αGM-CSF treated versus uninfected Ctrl Ab treated mice (middle), infected αGM-CSF treated versus infected Ctrl Ab treated mice (right) (Supplementary Data 4). Volcano plots, numbers and colour relate to genes that have an adjusted *P* value < 0.05. Blue, significantly down-regulated; red, significantly up-regulated; grey, non-differentially expressed. *P* values were adjusted for multiple testing using the Benjamini-Hochberg method. Heatmaps: gene expression values were averaged and scaled across the row to indicate the number of standard deviations above (red) or below (blue) the mean, denoted as row Z-score; shown for individual lung samples from uninfected Ctrl Ab (light green) or αGM-CSF (light purple) and infected Ctrl Ab (dark green) or αGM-CSF (dark purple) treated mice. Dendrogram shows unsupervised hierarchical clustering of genes.

Supplementary Figure 5

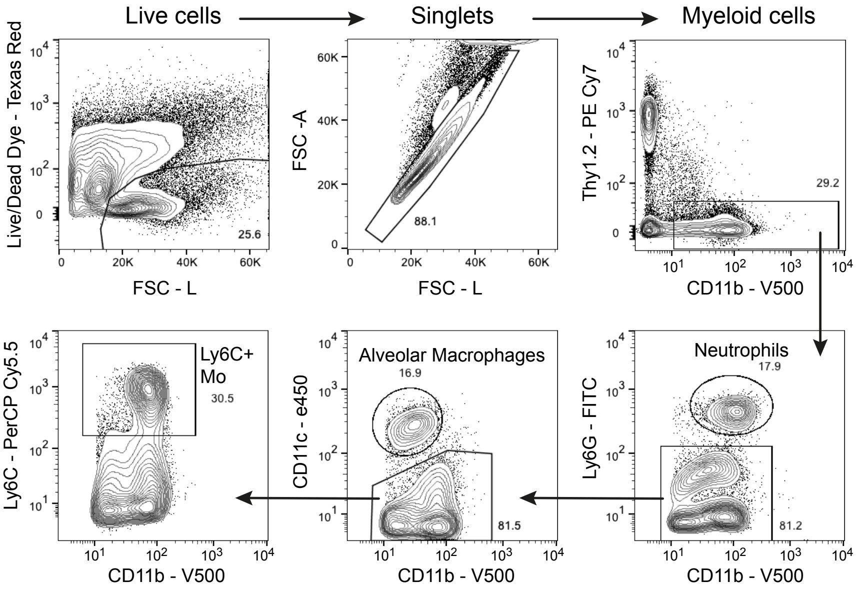

Supplementary Figure 5. GM-CSF regulates lung myeloid cell numbers during *M. tuberculosis* infection. WT mice were infected and treated with Ctrl Ab or αGM-CSF as in Fig. 1a. Lung cell suspensions were prepared and stained for detection of myeloid cells. Gating strategy for quantification of neutrophils ($CD11b^{+}Ly6G^{+}$), $Ly6C^{+}$ monocytes ($CD11b^{+}Ly6G^{-}Ly6C^{+}$) and alveolar macrophages ($CD11b^{low}CD11c^{+}$) is shown.

Supplementary Figure 6

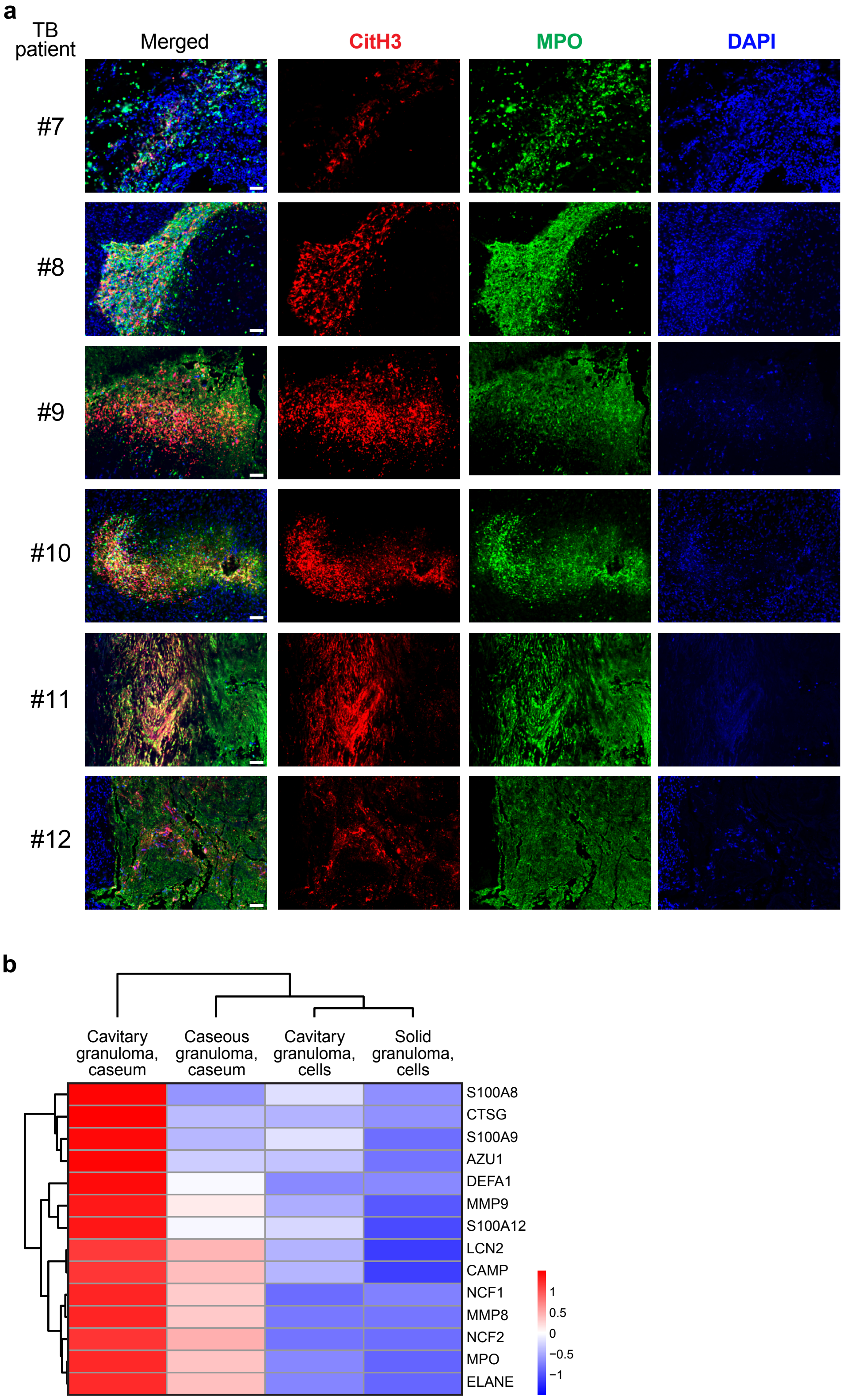

Supplementary Figure 6. NETs are detected in necrotic lung lesions of human pulmonary TB. a, Formalin-fixed paraffin embedded lung sections from a total of thirteen patients with pulmonary TB (1 section/ TB patient; Supplementary Table 1) were labelled with antibodies specific for citrullinated histone H3 (citH3; red) MPO (green) and DAPI (blue). All thirteen slides were stained and scanned together in the same experiment. NETs, visualized by colocalization of citH3 and DAPI staining (merged images, left), are shown for 6 TB patients. Scale bars, 50 µm. **b,** Unsupervised-hierarchical-clustering-based heatmap of z-scores of log2-transformed label-free quantification (LFQ) protein intensities quantified in lung TB granulomas samples (cavitary granuloma, caseum or cells; caseous granuloma, caseum; solid granuloma, cells) by Marakalala *et al.*[1], for the indicated neutrophil-associated proteins.

Supplementary Figure 7

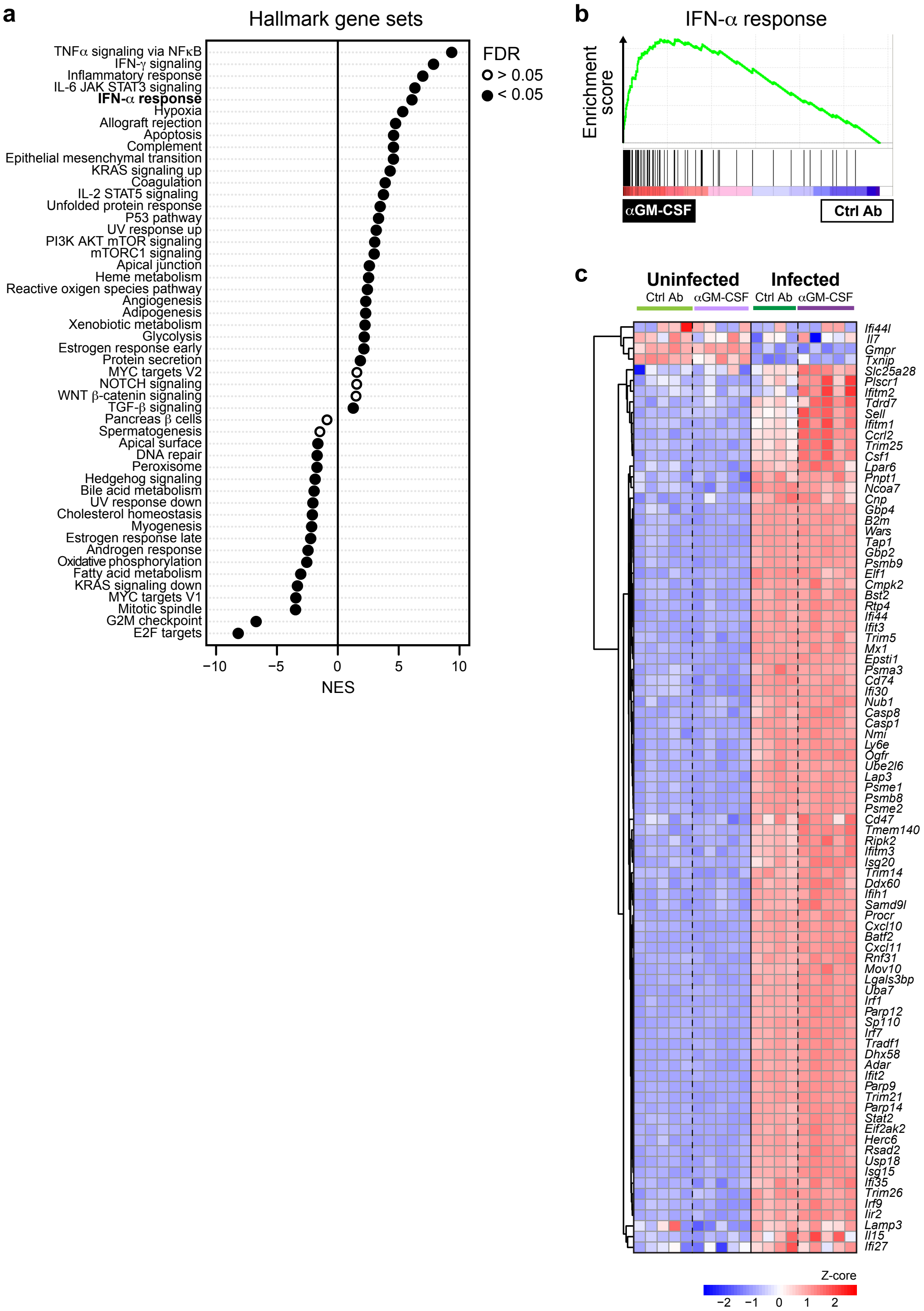

Supplementary Figure 7. Enhanced expression of genes associated with type I IFN-responses in *M. tuberculosis* infected lungs upon GM-CSF blockade. a, GSEA of hallmark gene sets from the Molecular Signatures Database of the Broad

Institute, showing the normalized enrichment scores (NES) for lung RNA-Seq data from infected αGM-CSF versus infected Ctrl Ab treated mice described in Supplementary Fig. 4. **b, c,** GSEA enrichment plot (**b**) and heatmap of genes (**c**) in the IFN-α response pathway. Gene expression values were averaged and scaled across the row to indicate the number of standard deviations above (red) or below (blue) the mean, denoted as row Z-score; shown for individual lung samples from uninfected Ctrl Ab (light green) or αGM-CSF (light purple) and infected Ctrl Ab (dark green) or αGM-CSF (dark purple) treated mice. Dendrogram shows unsupervised hierarchical clustering of genes.

Supplementary Table 1. TB patients

Patient ID	SH-TBL project patient's code	Sex	Age	BMI	Smoking (0=no, 1=yes)	Alcohol-consume (0=no, 1=yes)	Diabetes (0=no, 1=yes)	HCV (0=no, 1=yes)	Patient_history	Drug-resistance	Presence of TB symptoms (0=no, 1=yes)	Lesion size (in mm, diameter)	Presence Infiltrates X-ray (0=no, 1=yes)	NETs (0=no, 1=yes)
#1	SHTBL-013	male	16	25.5	0	0	0	0	new patient	MDR	0	30	0	1
#2	SHTBL-008	female	28	20	0	0	0	0	new patient	MDR	0	40	0	1
#3	SHTBL-005	female	25	19.1	0	0	0	0	new patient	MDR	0	30	0	1
#4	SHTBL-003	male	22	31.7	0	1	0	0	new patient	XDR	0	30	0	1
#5	SHTBL-034	male	40	30.41	1	0	0	1	relapse	DS	1	40	0	1
#6	SHTBL-023	female	22	18.4	0	0	0	0	relapse	XDR	0	31	0	1
#7	SHTBL-033	female	31	27.94	0	0	0	0	relapse	XDR	1	30	0	1
#8	SHTBL-001	male	32	23.1	1	1	0	0	new patient	DS	1	30	0	1
#9	SHTBL-002	male	61	24.2	1	1	1	0	relapse	DS	1	35	1	1
#10	SHTBL-011	male	50	22.5	0	0	1	0	new patient	MDR	0	35	0	1
#11	SHTBL-028	male	47	19.79	0	0	0	0	new patient	XDR	0	32	0	1
#12	SHTBL-009	female	36	32	0	0	0	0	relapse	XDR	0	38	0	1
#13	SHTBL-004	male	37	21.5	1	1	0	1	relapse	MDR	0	30	1	0

Abbreviations: DS, drug-sensitive; MDR, multi drug-resistant; XDR, extensively drug-resistant.

SUPPLEMENTARY REFERENCES

1. Marakalala, M.J. *et al.* Inflammatory signaling in human tuberculosis granulomas is spatially organized. *Nat Med* **22**, 531-538 (2016).
