# Supplementary material for: Type I IFN exacerbates disease in tuberculosis-susceptible mice by inducing neutrophil-mediated lung inflammation and NETosis
Source: Nat Commun. 2020 Nov 4;11:5566. doi: 10.1038/s41467-020-19412-6 (PMC7643080; doi:10.1038/s41467-020-19412-6)
Supplement: Supplementary file 3 — Descriptions of Additional Supplementary Files [file 41467_2020_19412_MOESM3_ESM.pdf]

Descriptions of Additional Supplementary Files

Supplementary Data 1

Description: Histopathological scoring from lung sections. Mice were infected and/or treated as in Fig. 1a. H&E stained lung sections were scored for histological parameters and relative lesion burden and data are shown separated in eleven tabs depicting individual experiments relative to Fig. 1 (five independent experiments), Fig. 6 (four independent experiments) and Fig. 9 (two independent experiments).

Supplementary Data 2

Description: Differently expressed genes in the blood. WT mice were infected and/or treated as in Fig. 1a. Blood was collected from infected and uninfected mice for RNA-Seq analysis (n= 4-5 mice/group). Differently expressed genes are shown separated in three tabs for comparisons: Infected Ctrl Ab versus Uninfected Ctrl Ab (related to Supplementary Fig. 2c, left panel), Uninfecetd aGM-CSF versus Uninfected Ctrl Ab (related to Supplementary Fig. 2c, middle panel), Infected aGM-CSF versus Uninfected Ctrl Ab (related to Supplementary Fig. 2c, right panel).

Supplementary Data 3

Description: Modular analysis of mouse blood RNA-seq data. WT mice were infected and/or treated as in Fig. 1a. Blood was collected from infected and uninfected mice for RNA-Seq analysis (n= 4-5 mice/group). Mouse blood data tested in human blood TB modules, from Singhania et al. 2018, separated in three tabs depicting: mouse blood group average - read counts per group in the mouse blood RNA-seq dataset for genes included in each human blood TB module; mouse blood individual - read counts for all individual samples in the mouse blood RNA-seq dataset for genes included in each human blood TB module; enrichment and FDR - module name, enrichment score and FDR for comparisons Infected Ctrl Ab versus Uninfected Ctrl Ab and Infected aGM-CSF versus Uninfected Ctrl Ab. Related to Fig. 2a.

Supplementary Data 4

Description: Differently expressed genes in the lung. WT mice were infected and/or treated as in Fig. 1a. Lungs were collected from infected and uninfected mice for RNA-Seq analysis (n= 4-5 mice/group). Differently expressed genes are shown separated in three tabs for comparisons: Infected Ctrl Ab versus Uninfected Ctrl Ab (related to Supplementary Fig. 4c, left panel), Uninfecetd aGM-CSF versus Uninfected Ctrl Ab (related to Supplementary Fig. 4c, middle panel), Infected aGM-CSF versus Uninfected Ctrl Ab (related to Supplementary Fig. 4c, right panel).

Supplementary Data 5

Description: Modular analysis of mouse lung RNA-seq data. WT mice were infected and/or treated as in Fig. 1a. Lungs were collected from infected and uninfected mice for RNA-Seq analysis (n= 4-5 mice/group). Mouse lung data tested in mouse lung disease modules, from Singhania et al. 2019, separated in three tabs depicting: mouse lung group average - read counts per group in the mouse lung RNA-seq dataset for genes included in each mouse lung disease module; mouse lung individual - read counts for all individual samples in the mouse lung RNA-seq dataset for genes included in each

mouse lung disease module; enrichment and FDR - module name, enrichment score and FDR for comparisons Infected Ctrl Ab versus Uninfected Ctrl Ab and Infected aGM-CSF versus Uninfected Ctrl Ab. Related to Fig. 2c.
