# Supplementary material for: Type I IFN exacerbates disease in tuberculosis-susceptible mice by inducing neutrophil-mediated lung inflammation and NETosis
Source: Nat Commun. 2020 Nov 4;11:5566. doi: 10.1038/s41467-020-19412-6 (PMC7643080; doi:10.1038/s41467-020-19412-6)
Supplement: Supplementary file 9 — Reporting Summary [file 41467_2020_19412_MOESM9_ESM.pdf]

nature research

Corresponding author(s): Lucia Moreira-Teixeira

Last updated by author(s): Sep 24, 2020

# Reporting Summary

Nature Research wishes to improve the reproducibility of the work that we publish. This form provides structure for consistency and transparency in reporting. For further information on Nature Research policies, see our Editorial Policies and the Editorial Policy Checklist.

## Statistics

For all statistical analyses, confirm that the following items are present in the figure legend, table legend, main text, or Methods section.

| n/a | Confirmed | |
|---|---|---|
| ☐ | ☒ | The exact sample size (*n*) for each experimental group/condition, given as a discrete number and unit of measurement |
| ☐ | ☒ | A statement on whether measurements were taken from distinct samples or whether the same sample was measured repeatedly |
| ☐ | ☒ | The statistical test(s) used AND whether they are one- or two-sided<br>*Only common tests should be described solely by name; describe more complex techniques in the Methods section.* |
| ☐ | ☒ | A description of all covariates tested |
| ☐ | ☒ | A description of any assumptions or corrections, such as tests of normality and adjustment for multiple comparisons |
| ☐ | ☒ | A full description of the statistical parameters including central tendency (e.g. means) or other basic estimates (e.g. regression coefficient) AND variation (e.g. standard deviation) or associated estimates of uncertainty (e.g. confidence intervals) |
| ☐ | ☒ | For null hypothesis testing, the test statistic (e.g. *F*, *t*, *r*) with confidence intervals, effect sizes, degrees of freedom and *P* value noted<br>*Give P values as exact values whenever suitable.* |
| ☒ | ☐ | For Bayesian analysis, information on the choice of priors and Markov chain Monte Carlo settings |
| ☒ | ☐ | For hierarchical and complex designs, identification of the appropriate level for tests and full reporting of outcomes |
| ☒ | ☐ | Estimates of effect sizes (e.g. Cohen's *d*, Pearson's *r*), indicating how they were calculated |

*Our web collection on statistics for biologists contains articles on many of the points above.*

## Software and code

Policy information about availability of computer code

Data collection

Histology images were obtained using cellSens Entry (v1.18) and OLYMPUS OlyVIA (v2.9).

Flow cytometry data were acquired using Summit software v4.4.0.

Software used for RNA-seq preprocessing:
FastQC (v0.10.1),
MultiQC (v1.6),
Trimmomatic (v0.36),
RSEM package (v1.3.30),
STAR alignment algorithm (v2.5.2a),
Ensembl mouse GRCm.38.89.

Data analysis

No custom code or algorithm was used to generate results that are reported in this study. Only publicly available data analysis tools were used. All tools, including parameters that were used where necessary, are provided in the 'Methods' section.
These tools included:
R (v3.4.3)
DESeq2 (v1.18.1),
pheatmap (v1.0.12),
ggplot2 (v3.3.2),
WGCNA (v1.68),
bioMart (v2.38.0),
QuSAGE (v2.18.1),

Broad’s GSEA software (v2.1.0),
MSigDB (v6),
Ward's algorithm,
CIBERSORT (v1.04).

Other data analysis tools:
FlowJo software (v10.3),
GraphPad Prism (v8.4.3) for macOS,
Fiji version 2.0.0 and Qupath 0.2.2.

For manuscripts utilizing custom algorithms or software that are central to the research but not yet described in published literature, software must be made available to editors and reviewers. We strongly encourage code deposition in a community repository (e.g. GitHub). See the Nature Research guidelines for submitting code & software for further information.

## Data

Policy information about availability of data

All manuscripts must include a data availability statement. This statement should provide the following information, where applicable:

- Accession codes, unique identifiers, or web links for publicly available datasets
- A list of figures that have associated raw data
- A description of any restrictions on data availability

The materials, data and any associated protocols that support the findings of this study are available from the corresponding author upon request. The RNA-sequencing datasets have been deposited in the NCBI Gene Expression Omnibus (GEO) database with the primary accession number GSE141207. Publicly available datasets used in this study include GSE109125 (sorted cells from Immunological Genome Project).

# Field-specific reporting

Please select the one below that is the best fit for your research. If you are not sure, read the appropriate sections before making your selection.

☒ Life sciences ☐ Behavioural & social sciences ☐ Ecological, evolutionary & environmental sciences

For a reference copy of the document with all sections, see nature.com/documents/nr-reporting-summary-flat.pdf

# Life sciences study design

All studies must disclose on these points even when the disclosure is negative.

| | |
|---|---|
| Sample size | No sample size calculation was performed. Animal sample estimates were determined using previous studies and/or pilot studies using 3-5 animals per group and guided by the 3R principle.<br>References: Gonzalez-Juarrero, M. et al. Disruption of granulocyte macrophage-colony stimulating factor production in the lungs severely affects the ability of mice to control Mycobacterium tuberculosis infection. J Leukoc Biol 77, 914-922 (2005); Moreira-Teixeira, L. et al. Mouse transcriptome reveals potential signatures of protection and pathogenesis in human tuberculosis. Nat Immunol 21, 464-476 (2020). |
| Data exclusions | Blood and lung RNA-seq samples from one C57Bl/6 mouse infected with HN878 and treated with anti-GM-CSF mAbs were excluded as they were found to be outliers during quality check. Exclusion criteria were not pre-established. |
| Replication | Biological replicates were included in all experiments, performed at least twice using independent infections. Where representative data are shown, the experimental findings were reproduced successfully in all independent experiments with similar results showed. Number of independent experiments for each figure is indicated in the figure legend. |
| Randomization | Animals were age and sex matched between experimental groups in order to account for covariates. The present study does not involve experiments other than those involving animals. |
| Blinding | Blinding was performed during H&E analysis of lung samples, with each sample scored as a consensus by three board-certified veterinary pathologists blinded to the groups. Blinding was also performed for NET area calculation. Blinding was not possible during sample collection and other analyses in the animal studies, as the investigators performing the experiments were the ones analyzing them. |

# Reporting for specific materials, systems and methods

We require information from authors about some types of materials, experimental systems and methods used in many studies. Here, indicate whether each material, system or method listed is relevant to your study. If you are not sure if a list item applies to your research, read the appropriate section before selecting a response.

## Materials & experimental systems

| n/a | Involved in the study | |
|---|---|---|
| ☐ | ☒ | Antibodies |
| ☒ | ☐ | Eukaryotic cell lines |
| ☒ | ☐ | Palaeontology and archaeology |
| ☐ | ☒ | Animals and other organisms |
| ☐ | ☒ | Human research participants |
| ☐ | ☒ | Clinical data |
| ☒ | ☐ | Dual use research of concern |

## Methods

| n/a | Involved in the study | |
|---|---|---|
| ☒ | ☐ | ChIP-seq |
| ☐ | ☒ | Flow cytometry |
| ☒ | ☐ | MRI-based neuroimaging |

# Antibodies

Antibodies used

Antibodies used in this study are as follows:

Monoclonal antibodies (mAbs) used for in vivo experiments:
anti-mouse-GM-CSF mAb (clone MP1-22E9, Lot A1102882, gift from DNAX (now Merck, USA); Lot 683718A1 from BioXCell: BE0259);
anti-mouse-IFN-g mAb (clone XMG1.2, BE0055;  Lot 5543-3/5543/0715) from BioXCell;
anti-mouse-Ly6G mAb (clone 1A8, BE0075; Lot 695418J3) from BioXCell;
anti-mouse-IFNAR1 mAb (clone MAR1-5A3, Prod. No. I-401; Lot 0419L555) from Leinco Technologies, Inc.

Isotype control mAb:
rat IgG2a, clone GL117, Lot A1102882, gift from DNAX (now Merck, USA);
rat IgG1, clone GL113, Lot A2022916, gift from DNAX (now Merck, USA);
rat IgG2a, clone 2A3, BE0089; Lot 71671801, from BioXCell.

Flow cytometry:
anti-FcgRI/FcgRII (anti-CD16/CD32; clone 24G2, Harlan: custom order),
anti-Thy1.2 (clone 53-2.1, eBioscience: 25-0902-82),
anti-Ly6G (clone 1A8, BD: 561105),
anti-Ly6C (clone HK1.4, eBioscience: 45-5932-82),
anti-CD11c (clone HL3, BD: 560521),
anti-CD11b (clone M1/70, BD: 562127),
anti-CD3 (clone 145-2C11, eBioscience: 17-0031-82),
anti-CD4 (clone RM4-5, eBioscience: 48-0042-82; 15-0042-82/BD: 560470),
anti-CD8 (clone 53-6.7, BD: 553032; 560776 / clone Ly-2, eBioscience: 12-0081-82),
anti–IFN-g (clone XMG1.2, eBioscience: 48-7311-82) or isotype control (clone eBRG1, eBioscience: 48-4301-82).

For in vitro culture:
anti-CD28 (clone 37.51, Harlan: custom order).

For immunofluorescence detection of NETs:
rabbit anti-mouse antibodies directed against citrullinated histone H3 (ab5103 from Abcam);
goat anti-mouse antibodies directed against MPO (AF3667, R&D systems);
secondary donkey anti-rabbit IgG antibodies conjugated with Alexa Fluor 568 (A10042, Invitrogen);
secondary donkey anti-goat IgG antibodies conjugated with Alexa Fluor 488 (A11045, Invitrogen).

Validation

Monoclonal antibodies (mAbs) used for in vivo experiments:
All mAbs for in vivo experiments were validated by the manufacturer for the reactivity species: mouse and application: in vivo neutralization/blocking.
Anti-mouse-GM-CSF mAb was further validated in house using GM-CSF-deficient mice, with no unspecific effects observed.
The XMG1.2 monoclonal antibody reacts with mouse IFNγ and it was further validated in house with observed increased susceptibility to Mycobacterium tuberculosis infection as reported in the literature.
The 1A8 monoclonal antibody reacts specifically with mouse Ly6G with no reported cross reactivity with Ly6C; and it was further validated in house with observed decreased in circulating neutrophils following in vivo treatment.
Clone MAR1-5A3 has a short half-life due to the rapid recycling of cells that express the IFNAR1 receptor. In order to block function In vivo, continual blocking of all compartments is necessary. Therefore, a large loading dose is necessary to saturate all In vivo binding sites and should be maintained to ensure binding site saturation.

Flow cytometry:
All flow cytometry antibodies used are commercially available and were validated by the manufacturer for the reactivity species: mouse and application: flow cytometry.

For in vitro culture:

anti-CD28 was certified for cell culture by the provider (reactivity species: mouse).

For immunofluorescence detection of NETs:
Primary antibodies used for immunofluorescence detection of NETs are commercially available and were validated by the respective manufacturer for the reactivity species: human and mouse. Antibodies directed against citrullinated histone H3 and MPO were further validated in house by staining of positive and negative mouse tissue samples. Controls were also stained with secondary antibody only, and nonspecific fluorescent staining was not detected when secondary antibodies were tested alone.

## Animals and other organisms

Policy information about studies involving animals; ARRIVE guidelines recommended for reporting animal research

| | |
|---|---|
| Laboratory animals | C3HeB/FeJ, C57BL/6, Ifngr1tm1Agt, Ifnar1tm1Agt, Ifnar1tm1Uka, and Tg(S100A8-cre,-EGFP)1Ilw mice were bred and housed in specific pathogen-free facilities at The Francis Crick Institute. Age matched (8 to 16 week-old) females were used in experiments. |
| Wild animals | The study did not involve wild animals. |
| Field-collected samples | The study did not involve samples collected from the field. |
| Ethics oversight | All protocols for breeding and experiments were performed in accordance with Home Office (U.K.) requirements and the Animal Scientific Procedures Act, 1986; or in accordance with recommendations of the European Union Directive 2010/63/EU and approved by Portuguese National Authority for Animal Health – Direção Geral de Alimentação e Veterinária (DGAV-Ref.0421/000/000/2016). |

Note that full information on the approval of the study protocol must also be provided in the manuscript.

## Human research participants

Policy information about studies involving human research participants

| | |
|---|---|
| Population characteristics | The human samples used for this study came from the collection obtained within the SH-TBL project (Supplementary Table 1; http://dx.doi.org/10.17632/knhvdbjv3r.1), led by the Experimental Tuberculosis Unit (UTE) and conducted in collaboration with the National Center for Tuberculosis and Lung Diseases of Georgia (NCTLD); and registered at the ClinicalTrials.gov database under code NCT02715271. Total of 13 TB patient: 5 females and 8 males; ages between 16 and 61 years old; TB drug sensitivity: 3 drug-sensitive (DS), 5 multi drug-resistant (MDR), and 5 extensively drug-resistant (XDR). |
| Recruitment | Patients undergoing therapeutical surgery for Pulmonary Tuberculosis (DS- and MDR/XDR-TB) indicated as per clinical routine, at NCTLD in Tbilisi, Georgia. Samples were selected according to the patient's drug resistance and maturation status of the lung TB lesion, with all drug resistance (DS, MDR and XDR) and maturation status (in terms of necrosis, fibrosis and calcification) being represented. |
| Ethics oversight | The project was reviewed and approved by both the Ethics Committee of the NCTLD (IRB00007705 NCTLD Georgia #1, IORG0006411) and the Germans Trias I Pujol Hospital (IGTP) ethics committee (EC: PI-16-171). Written informed consent was obtained for collection of biological material and data from all study participants before being enrolled. |

Note that full information on the approval of the study protocol must also be provided in the manuscript.

## Clinical data

Policy information about clinical studies
All manuscripts should comply with the ICMJEguidelines for publication of clinical research and a completedCONSORT checklist must be included with all submissions.

| | |
|---|---|
| Clinical trial registration | NCT02715271 |
| Study protocol | https://clinicaltrials.gov/ct2/show/NCT02715271?term=vilaplana&cond=tuberculosis&draw=2&rank=3 |
| Data collection | Samples were obtained within the Study of the Human Tuberculosis Lesions Project (SH-TBL project, registered at ClinicalTrials.gov NCT02715271), involving Georgian TB patients undergoing therapeutic surgery in the National Center for Tuberculosis and Lung Diseases (NCTLD) (Tbilisi, Georgia) between May 2016 and May 2018. All patients recruited received therapy according to national guidelines prior surgery and surgery was prescribed according to official guidelines' surgery recommendations. |
| Outcomes | Primary Outcome Measures:<br>1 - Description of Clinical and Epidemiological Data of the TB patients enrolled at baseline; descriptive analysis; data recorded in a spreadsheet created ad-hoc;<br>2 - Description of the Histopathological characteristics of the TB lesions of TB patients enrolled at baseline; descriptive analysis; data recorded in a spreadsheet created ad-hoc<br>3 - Obtention of a genic-proteomic profile of TB lesions tissues which correlate to the histopathology of the granulomas and/or the clinic-pathological features of TB patients at baseline; descriptive analysis, correlation with histopathological characteristics of TB lesions and with clinical and epidemiological data of TB patients<br><br>Secondary Outcome Measures :<br>1 - Change in Immunological responses at baseline; and at the moment of discharge, an average of at day 15 post-enrollment. Identification of biomarkers in blood at protein level in blood and urine; validation in tissue and blood of genic biomarkers secreted (ELISA, qPCR, immunohistochemistry). |

2 - Change in Health Quality of Life Measurements at baseline and through study completion, an average of 1 year; measured with Health Quality of Life Questionnaires.

## Flow Cytometry

### Plots

Confirm that:

- [x] The axis labels state the marker and fluorochrome used (e.g. CD4-FITC).
- [x] The axis scales are clearly visible. Include numbers along axes only for bottom left plot of group (a 'group' is an analysis of identical markers).
- [x] All plots are contour plots with outliers or pseudocolor plots.
- [x] A numerical value for number of cells or percentage (with statistics) is provided.

### Methodology

| | |
|---|---|
| Sample preparation | Cell homogenates prepared from harvested lungs from infected mice were stained according to manufacturer's instructions to exclude dead cells using a Live/Dead fixable red dead cell stain kit (Invitrogen). Cells were pre-treated for 10 min with anti-CD16/CD32 and then stained with Abs against specific extracellular markers to identify myeloid cells or lymphocytes.<br><br>For detection of IFN-g production, cells were restimulated ex vivo with M. tuberculosis tuberculin purified protein derivative (PPD; Statens Serum Institute) and anti-CD28 (clone 37.51, Harlan) for 20 h. Brefeldin A (Sigma-Aldrich) was added during the last 4 h. After extracellular staining, cells were fixed and treated with permeabilization buffer (BD) according to manufacturer's instructions and stained with anti–IFN-g or isotype control Abs.<br><br>All stained samples were fixed with stabilizing fixative (BD) and refrigerated in the dark overnight before being acquired. |
| Instrument | Samples were acquired on a CyAN ADP analyser (Dako, Ely, U.K.) in a Biosafety Level 3 facility. |
| Software | Data were acquired using Summit software (Cytomation) and analysed using FlowJo software (Tree Star). |
| Cell population abundance | Cell sorting was not performed in this study. |
| Gating strategy | Myeloid cell populations were analysed after exclusion of dead cells and Thy1.2+ cells; neutrophils were gated as CD11b +Ly6G+, Ly6C+ monocytes as CD11b+Ly6G-Ly6C+ and alveolar macrophages as CD11b low CD11c+.<br><br>T cell populations were analysed after exclusion of dead cells and gating on Thy1.2+CD3+ cells. |

- [x] Tick this box to confirm that a figure exemplifying the gating strategy is provided in the Supplementary Information.
